# Prevalence of diarrhea and water sanitation and hygiene (WASH) associated factors among children under five years in Lira City Northern Uganda: Community based study

**DOI:** 10.1371/journal.pone.0305054

**Published:** 2024-06-07

**Authors:** Brenda Auma, Marvin Musinguzi, Edward Ojuka, Eustes Kigongo, Raymond Tumwesigye, Walter Acup, Amir Kabunga, Bosco Opio

**Affiliations:** 1 Department of Community Health, Faculty of Public Health, Lira University, Lira, Uganda; 2 Department of Quality Assurance, Lira University, Lira, Uganda; 3 Department of Environmental Health and Disease Control, Faculty of Public Health, Lira University, Lira, Uganda; 4 Department of Nursing, Faculty of Nursing and Midwifery, Lira university, Lira, Uganda; 5 Department of Psychiatry, Faculty of Medicine, Lira University, Lira, Uganda; 6 Department of Epidemiology and Biostatistics, Faculty of Public Health, Lira University, Lira, Uganda; Debre Markos University, ETHIOPIA

## Abstract

**Background:**

Children under the age of five experience a significant disease burden from diarrheal illnesses. This poses a severe public health risk as the second leading cause of infant death worldwide, after pneumonia. Lira City in Uganda is one of the developing urban areas with limited information about the diarrheal disease among children under the age of 5 years. This study aimed to determine the prevalence and assess the water, sanitation and hygiene related factors associated with diarrheal diseases among children under five years in Lira City.

**Methods:**

The study was conducted among 492 care takers of children under the age of 5 years in Lira City between August 2022 and September 2022. Data was collected using an interviewer administered questionnaire and a multi-stage sampling was used to select study participants. Data was analyzed by bivariate and multivariate logistic regression using STATA version 17. P-value of < 0.05 was considered statistically significant.

**Results:**

Out of 541 participants, 492 responded. The majority of the respondents, 425(86.4%) were female, 146(29.7%) had children aged 1–12 months, 192 (39%) had primary level education, and 155(31.5%) were self-employed. The prevalence of diarrhea among children under five years was 130(26.4%) and the associated factors with diarrheal disease were children between 49–60 months old (AOR = 0.12, 95% *CI*: 0.03–0.39, P = 0.001), cleaning the latrine more times (AOR = 0.42, 95% *CI*: 0.22–0.81, P = 0.010) and not treating water (AOR = 1.84, 95% *CI*: 1.11–3.06, P = 0.018).

**Conclusion:**

There is high prevalence of diarrhea among children under 5 years of age. The study’s findings highlight the need for ongoing efforts to lower the prevalence of diarrheal illnesses among children under the age of five in Uganda’s emerging urban areas.

## Introduction

Children under the age of five experience a significant disease burden from diarrheal illnesses. According to United Nations International Emergency Fund (UNICEF), diarrheal illnesses pose a severe public health risk as the second leading cause of infant death worldwide, after pneumonia [1]. For instance, children under the age of five years account for approximately 63% of the global diarrhea burden [2]. Additionally, 1.7 billion cases of pediatric diarrheal infections are recorded annually globally, with an estimated 525,000 children under the age of five dying from these illnesses [1]. In Africa, Asia and South America for instance diarrhea kills one out of every eight children aged 5 and below [3].

The World Health Organization defines diarrhea as the passing of three or more loose or watery stools per day [4]. More so the WHO also classifies diarrhea into three clinical types: acute watery diarrhea is defined as the abrupt onset of 3 or more loose stools per day (24 hours) and lasts no longer than 14 days, acute bloody diarrhea (dysentery) and persistent diarrhea lasting 14 days or longer [4]. Diarrhea pauses serious health risks to children’s health because during episodes of diarrhea water and electrolytes are lost leading to profound dehydration. Failure to replace fluids puts the child at a heightened risk of shock and potential fatality [4]. Additionally, this vulnerability exposes the child to the increased likelihood of contracting other bacterial infections. The predominant causative agents for diarrhea cases in children under 5 years include Rotavirus, cryptosporidium, ETEC, and shigella [5]. Particularly, Rotavirus stands as the leading cause of diarrhea-related mortality in this age group, with 72.1% of cases attributed to contaminated water and 56% to inadequate sanitation [5, 6].

Diarrhea, on the other hand, is a highly preventable disease that can be avoided by having adequate water, practicing proper sanitation, and hygiene practices. For example, studies have shown that hand washing with soap at critical moments can save one out of every three diarrhea-related deaths [7–10]. However, despite of even the COVID-19 pandemic putting a spot light on the importance of WASH, Worldwide, 2.2 billion people still lack access to safe drinking water. More than half of the global population does not have access to safe sanitation. Three billion people do not have access to handwashing facilities with soap. Still, 673 million people practice open defecation [1]. Sub Saharan Africa has the lowest levels of access to WASH services, and this access is uneven depending on where people live (SSA sub regions and rural or urban areas) and their socioeconomic status [11].

In Uganda barely just 18% of the population nationally has access to a basic sanitation service [12]. Majority of the population in slum areas have unsafe modes of fecal matter disposal such as ‘flying toilets’ [13]; in Kampala the capital City of the country, only 6.5% of households had hand washing facilities at homes [14]. According to the Uganda demographic health survey, prevalence of diarrhea among children under five years in Uganda is 20% [15]. A cross national study in 2019 also reported the overall under-five death rate of 90 deaths per 1000 live births. Of these, 93% were attributable to diarrheal diseases [16]. Further investigation is required to comprehensively understand the factors associated with diarrheal disease in Uganda. However, existing evidence suggests that limited access to clean water, substandard sanitation conditions, insufficient hygiene practices, and prevalent malnutrition are significant contributors to the persistent high rates of diarrheal diseases in the country [17, 18]. Through the Ministry of health, ongoing initiatives to prevent and control diarrheal disease among children focus on vaccination, WASH promotion, and healthcare infrastructure improvements [19].

Northern Uganda accounted for 20–40% of the overall deaths [16]. However, the region was also severely affected by war for over 2 decades that disrupted the Health, social and economic systems hence hindering development [20]. Lira City is one of the cities located in Northern Uganda with a rapidly growing population and upcoming slums [21]. However, there is still limited data on WASH situation and its association with the prevalence of Diarrheal diseases among children under the age of 5 years.

This study therefore, aimed at determining the prevalence and assess the water, sanitation and hygiene related factors associated with diarrheal diseases among children under five years in Lira City. The results of this study are crucial in providing information regarding the causes of diarrheal diseases among children under the age of 5 years living in urban areas of low- and middle-income countries, such as Uganda. This is crucial for making informed decisions to lower the prevalence of diarrheal illness in urban environments.

## Materials and methods

### Study design and study area

This was a community-based cross-sectional study conducted among children under the age of 5 years in Lira City on working days from 10^th^ August 2022 to 15^th^ September 2022. Lira City is located in Northern Uganda in Lira district. Lira district is also located within Lango sub region and primarily populated by the Lango tribe. Lira City has a current population of 474,200 people living with an estimated 13,287 children under the age of 5 years in the City with in the East and West division [22]. The main source of water in the city is Uganda National Water and sewerage Co-operation and 356 bore holes with in the city. The drinking water coverage and the latrine coverage of the city are estimated at 86% and 95%, respectively. Hand washing is practiced by 70% of the population [21].

### Study population

All children under 5 years in households within Lira City, including both divisions; City West and City East divisions were considered as the source population. the target population was all the parents and care takers of children under the age of 5 years in Lira city. However, the study population was parents or caretakers of children under the age of 5 years from selected households in Lira city.

### Inclusion and exclusion criteria

The study included all caretakers aged 18 years and above caring for children below 5 years in Lira City, who consented to the study. Only children less than 5 years from the households where a parent or caretaker was present at the time of the study were included. In households were more than one child under five years existed, the eldest child was selected for inclusion into the study. The rationale behind this approach is to mitigate the clustering effect, wherein a respondent might provide identical information for more than one child. Additionally, the inclusion of older children is considered, as it enhances the potential for obtaining more comprehensive information from the respondent.

All caretakers aged 18 years and above caring for children below 5 years and had not spent adequate time (at least 1 week) with the child were excluded from this study. Also care takers who were critically ill and un able to provide information for our study were excluded from the study. Those who were not free to provide the required information were excluded. Additionally, all children under 5 years whose mothers or caretakers had not lived in Lira City for at least six months prior to conducting the study.

### Sample size determination and sampling technique

The Kish Leslie (1965) formula was used to estimate the sample size [23]. The sample size was given by Z = 1.96 at 95% confidence level, d = allowable error of 5%, p = 20% which is the national prevalence of diarrheal diseases among children under five years of age according to UDHS [15], we obtained a sample size of 492. After adjusting for a non-response rate of 10% and design effect of 2.0 due to multi-stage sampling, we obtained a sample size of 541 participants as follows:

$$\begin{array}{l} n=deff*Z^{2}*p\left(1-p\right)/d^{2}\\ n=2.0*1.96^{2}*0.20\left(1-0.20\right)/0.05^{2}=492\\ n=492+\left(492*10\%\right)=541\;\text{respondents} \end{array}$$

A multi stage sampling technique was used to select participants of the study. Parishes were selected using systematic random sampling. A parish is an administrative unit within a district. It is a sub-county division and represents a lower administrative level than a sub-county. Parishes are further divided into villages and are part of the decentralized system of governance in Uganda. villages were also selected using simple random sampling and households were also selected using simple random sampling with the help of Village leaders and Village health teams the teams had records of all the households with in the villages. The total number of participants to be obtained from the village was determined using proportionate by size. Households with children under the age of 5 years were extracted and sampling frames were generated. See Table 1.

**Table 1:** Showing the sampling techniques used to select caretakers of children under five years in Lira City Northern Uganda, 2022.

Division	Sub county (Systematic random sampling)	Village (simple random sampling)	Sample
East Division	Ireda East	Boke agali	80
		Ireda Agali	73
	Te-Obia	Town college	71
		Camp Swahili	65
West Division	Barapwo subcounty	Elwa	68
		Te-dam	31
	Amuca Sub county	Amin-yang	53
		Owiti	51

### Operational definitions

#### Diarrheal disease

Was defined as a disease that presents with a sign of passing out of watery stool 3 times in one day [4]. However, in this study, a child who had diarrhea had to have experienced 3 episodes of watery stool and this should have occurred with in the previous 2 weeks.

#### Sanitation

Was defined as a having access to facilities for the safe disposal of human waste (feces and urine) through practices such as cleaning the latrine and use of rubbish bin [9].

#### Hygiene

Was defined as behaviors that can improve cleanliness and lead to good health, such as frequent handwashing with soap and bathing [11].

#### Care takers

Were defined as anyone who was caring for a child below the age of 5 years. The care takers cold have been one of the parents, close relative, distant relative or maid. The person should have been taking care of the child 2 weeks prior to the study [24].

#### Water treatment

Was defined as any process that is done to make water safe to drink and use this includes boiling, filtering and use of chemicals such as chlorine tablets [25].

### Study variables

#### Dependent variable

The dependent variable was the occurrence of diarrheal illnesses in children under the age of five years in the two weeks before to the survey interview. For this purpose, the demographic health survey definition of diarrhea was used, which is defined as having three or more loose or liquid bowel movements in a 24-hour period, as reported by the child’s mother or caregiver, at any time during the two weeks prior to the interview, and this was assessed by asking mothers/caregivers if their children had diarrhea in the two weeks prior to data collection. The result was a binary response with yes or no answer.

#### Independent variable

The independent variables included socio-economic and demographic characteristics of respondents, such as the mother or caretaker’s age, the sex of the child, marital status, level of income, and family size. Water-related factors encompassed the water source, collection, storage, and drinking water. Environmental sanitation factors involved the availability of a latrine, the type of toilet facility, the main floor material, and child stool disposal. Hygiene factors included the availability of hand-washing facilities, the availability of soap, hand-washing at critical points, and food handling practices.

#### Data collection tool and quality assurance

Data was collected using an interviewer administered questionnaire. The questionnaire was developed from several WASH tools including UNHCR WASH assessment tools and literature review from related studies [10, 26, 27]. The tool was sectioned into: socio-demographics, water factors, sanitation factors, and hygiene factors including feeding practices. The tool was then translated into Lango language by a WASH team from Plan International and pretested in Kole district one of the neighboring districts of Lira district. The data collection tool underwent a validation process, with the content validity index yielding a score of 7.6. Furthermore, the tool demonstrated a high level of reliability, as indicated by a coefficient of 8.2. Five research assistants fluent in Lango language were recruited, trained and used to collect under super vision of the investigators.

#### Data management and analysis

Data was entered into Microsoft Excel and cleaned. The validated data was then exported to STATA version 17 for analysis. Descriptive statistics were used to summarize the socio-demographic data and also to analyze for the prevalence of diarrheal disease. Prevalence of diarrheal disease was calculated as the percentage of the care takers children who had experienced 3 or more episodes of diarrhea cases of the total analyzed. Bivariate logistic regression analysis was done using bivariate logistic regression and crude odds ratios (COR) at 95% *CI* were used. All variables that were plausible considering prior knowledge and those that gave a *p*-value ≤0.2 at the bivariate logistic regression analysis were considered for multivariate logistic regression analysis.

Multivariate logistic regression analysis was used to identity significant independent predictors for Diarrheal disease. Independence of errors was verified through the evaluation of residuals to ensure that no discernible patterns or trends were present. Checks for multicollinearity involved calculating variance inflation factors (VIF) for each independent variable, ensuring that no VIF exceeded a predetermined threshold, indicating the absence of perfect multicollinearity. Identification and handling of outliers and influential observations were performed through thorough scrutiny of studentized residuals and leverage statistics. Homoscedasticity of errors was assessed by examining plots of residuals against predicted values to confirm constant variance.

Adjusted odds ratios (AOR) at *95% CI* were calculated for as measures of association at multivariate logistic regression. Variables with the *p*-value of p < 0.05 was considered to be statistically significant with the outcome variable.

#### Ethical considerations

Ethical approval to conduct the study was obtained from Gulu University Research Ethics Committee GUREC-2022-249. Written informed consent was obtained from the care takers of the children under the age of 5 years. Throughout the study, confidentiality of participants’ information was maintained through password protection of softcopy data and the use of codes for names and key information such as names. Interviews were done in places chosen by the participants at their homes, preferably without any other people. Respect for the participants as outlined in the Helsinki Declaration was maintained.

## Results

Out of the 541 participants 492 were able to participate in the study resulting into a response rate of 91%. The prevalence of diarrhea among children under the age of 5 years was 26.4% in lira city (95% *CI*: 0.23–0.31).

### Demographic factors associated with diarrheal disease among children under five years in Lira City Northern Uganda

Table 2 shows that the majority of the respondents, comprising 86.4% (425) females. Additionally, 29.7% (146) reported having children aged 1–12 months. A significant number 377(76.6%) identified as Christians, with 39% (192) having primary level education, and 31.5% (155) being self-employed. Furthermore, 48.2% (237) were in the age range of 25–35 years, and 65.9% (324) had family sizes between 1–5 members. More so, children between 47–60 months of age was significantly associated with Diarrheal disease among children under the age of 5 years at bivariate logistic regression analysis (COR = 0.12, 95% *CI*: 0.03–0.39, p = 0.001).

**Table 2:** Demographic factors associated with diarrheal disease among children under five years in Lira City Northern Uganda, 2022.

Variable	Frequency N (%)	Diarrhea		COR(*95%CI*)	P value
		No N(%)	Yes N(%)		
**Diarrhea**		**362(73.6)**	**130(26.4)**		
**Sex**					
Male	67(13.6)	52(77.6)	15(22.4)	1.00	
Female	425(86.4)	310(73)	115(27)	1.30(0.70–2.37)	0.421
**Age of child**					
1–12 months	146(29.7)	94(64.3)	52(35.7)	1.00	
13–24 months	101(20.5)	74(73.2)	27(26.8)	0.65(0.37–1.15)	0.142
25–36 months	116(23.6)	86(74.1)	30(25.9)	0.63(0.36–1.07)	0.092
37–48 months	82(16.7)	64(78)	18(22)	0.50(0.27–0.95)	0.033
49–60 months	47(9.6)	44(93.6)	3(6.4)	0.12(0.03–0.39)	0.001**
**Sex of child**					
Male	208(42.3)	153(74)	55(26)	1.00	
Female	284(57.7)	209(74)	75(26)	0.99(0.67–1.50)	0.993
**Religious affiliation**					
Muslim	26(5.3)	19(73)	7(27)	1.00	
Christian	377(76.6)	289(76.7)	88(23.3)	0.83(0.34–2.03)	0.678
Traditional/Indigenous religions	89(18.1)	54(60.6)	35(39.4`)	1.75(0.67–4.62)	0.251
**Education level**					
Primary	192(39)	130(68)	62(32)	1.00	
Secondary	168(34.1)	130(77.3)	38(30)	0.61(0.38–0.98)	0.042
Tertiary	100(20.3)	77(77)	23(23)	0.62(0.35–1.09)	0.099
None	32(6.5)	25(78.1)	7(21.9)	0.58(0.24–1.43)	0.241
**Employment status**					
Employed	41(8.3)	26(63)	15(37)	1.00	
Self employed	155(31.5)	119(77)	36(23)	0.52(0.25–1.09)	0.086
Unemployed	296(60.2)	217(73.3)	79(26.7)	0.63(0.31–1.25)	0.188
**Age of care taker**					
< 18 years	61(12.4)	48(78.6)	13(21.4)	1.00	
19–24	153(31.1)	102(66.6)	50(33.4)	1.80(0.89–3.64)	0.097
25–35	237(60.2)	176(74.2)	61(25.8)	1.20(0.64–2.52)	0.476
36–45	26(5.3)	22(84.6)	4(15.4)	0.67(0.19–2.29)	0.525
46 and above	15(3.0)	13(86.6)	2(13.4)	0.56(0.11–2.84)	0.491
**Family size**					
1–5	342(65.9)	246(76)	77(24)	1.00	
6–10	158(32.1)	111(70.2)	46(29.8)	1.32(0.86–2.03)	0.199
11>	10(2)	4(40)	6(60)	4.80(1.31–17.42)	0.17

*Level of significance at p<0.05,

**level of significance at p<0.001, p<0.05

### Water related factors associated with diarrheal disease among children under five years in Lira City Northern Uganda

Table 3 shows that majority of respondents accessed water from taps 51.4% (254). Boiling was the primary water treatment method 30.7% (151). Earthen pots were the preferred storage method 70.7% 348. In daily water usage, 50.1% (246) consumed 41–100 liters. Only not treating drinking water was significantly associated with diarrheal disease (COR = 2.01, 95% *CI*: 1.29–3.41, p = 0.005). Other variables were not significantly associated with diarrheal disease.

**Table 3:** Water hygiene and sanitation factors associated with diarrheal disease among children under five years in Lira City Northern Uganda.

Variable	Frequency %(N)	Diarrheal disease		COR(95%CI)	P Value
		No N(%)	Yes N(%)		
**Water source**					
Borehole	149(30.3)	108(72.5)	41(27.5)	1.00	
Tap	254(51.4)	197(77.6)	57(22.4)	0.76(0.47–1.21)	0.252
Shallow well	27(5.5)	17(63)	10(37)	1.53(0.65–3.66)	0.318
Protected Spring	62(12.6)	40(64.5)	22(35.5)	1.44(0.77–2.72)	0.251
**Water Treatment**					
Boiling	151(30.7)	124(82.1)	27(17.9)	1.00	
Filtering	27(5.5)	22(81.4)	5(18.6)	1.04(0.36–3.00)	0.937
Chemical treatment	18(3.7)	13(72.2)	5(27.8)	1.22(0.58–5.37)	0.316
No treatment	296(60.2)	203(68.5)	93(31.5)	2.01(1.29–3.41)	0.003**
**Water Storage**					
Earthen Pot	348(70.7)	259(72.3)	99(27.7)	1.00	
Refrigerator	35(7.1)	26(81.3)	6(18.8)	0.60(0.24–1.51)	0.281
Jerry can	95(19.3)	69(74.2)	24(25.8)	0.90(0.54–1.52)	0.722
Don’t store	14(2.9)	8(88.9)	1(11.1)	0.32(0.04–2.64)	0.295
**Liters of water used per day**					
<20	14(2.9)	11(84.6)	3(15.4)	1.00	
21–40	23(4.7)	20(6.6)	3(93.4)	0.55(0.09–3.20)	0.506
41–100	246(50.4)	184(74.7)	62(25.3)	1.24(0.33–4.57)	0.751
>100	208(42.4)	146(70.1)	62(29.9)	1.55(0.41–5.77)	0.508

*level of significance at p<0.05,

**level of significance at p<0.001, p<0.05

### Hygiene related factors associated with diarrheal disease among children under five years in Lira City Northern Uganda

Table 4 shows majority of respondents 83.7% (412) reported not washing hands. Regarding washing the child’s bottom after defecation, the majority 65% (320) reported always doing it. For washing hands after handling feces, a significant majority 94.5% (465) indicated compliance, while 5.5% did not. Similarly, the majority (82.1%) reported washing hands before feeding their child. However, none of the factors was significantly associated with diarrheal disease.

**Table 4:** Hygiene related factors associated with diarrheal disease among children under five years in Lira City Northern Uganda.

Variable	Frequency %(N)	Diarrheal disease		COR(*95%CI*)	P-Value
		No N(%)	Yes N(%)		
**Hand washing**					
Yes	80(16.3)	58(72.5)	22(27.5)	1.00	
No	412(83.7)	304(73.8)	108(26.2)	0.93(0.55–1.60)	0.94
**Washing the child’s bottom after defecation**					
Always	320(65)	237(74.1)	83(25.9)	1.00	
Sometimes	136(27.6)	96(70.6)	40(29.4)	1.19(0.76–1.80)	0.445
Rarely	27(5.5)	21(77.8)	6(22.2)	0.81(0.31–2.09)	0.672
Never	9(1.8)	8(88.9)	1(11.1)	0.35(0.04–2.89)	0.335
**Wash hands after handling feces**					
Yes	465(94.5)	339(72.9)	126(27.1)	1.00	
No	27(5.5)	23(85.2)	4(14.8)	0.46(0.15–1.37)	0.169
**Wash hands before feeding child**					
Yes	404(82.1)	301(74.5)	103(25.5)	1.00	
No	88(17.9)	61(70.1)	26(29.9)	0.61(0.31–1.31)	0.225

*level of significance at p<0.05, **level of significance at p<0.001, p<0.05

### Sanitation related factors associated with diarrheal disease among children under five years in Lira City Northern Uganda

Table 5 shows that majority of the respondent had rubbish pits in their homes 75.1%(369), 85.2 (420%) had latrines, 85.7 (420) had clean wash rooms, 37.8% (172) cleaned their latrines more than 7 times a week and about 86%(423). Only cleaning the latrine more than 7 times in a week had a significant relationship with Diarrheal disease (COR = 0.45, 95% *CI*: 0.24–0.83, p = 0.011).

**Table 5:** Sanitation related factors associated with diarrheal disease among children under five years in Lira City Northern Uganda.

Variable	Frequency N(%)	Diarrheal disease		COR(*95%CI*)	P Value
		No N(%)	Yes N(%)		
**Rubbish Disposal**					
Rubbish pit	369(75.1)	278(75.3)	91(24.7)	1.00	
Open Dumping	75(15.3)	49(65.3)	26(34.7)	1.62(0.95–2.75)	0.075
Stored in vessels	36(7.3)	27(75)	9(25)	1.01(0.46–2.24)	0.964
None	11(2.2)	8(72.7)	3(27.3)	1.14(0.29–4.40)	0.843
**Availability of Latrine**					
Yes	419(85.2)	113(26.9)	306(73.1)	1.00	
No	73(14.8)	17(23.2)	56(76.8)	0.82(0.46–1.48)	0.511
**Clean WASH Rooms**					
Clean	420(85.7)	309(73.6)	111(26.4)	1.00	
Not clean	70(14.3)	52(74.3)	18(25.7)	0.96(0.54–1.71)	0.9
**Number of times latrine is cleaned**					
Less than 7 times a week	69(15.1)	43(62.3)	26(37.7)	1.00	
7 times a week (every day)	215(47.1)	154(71.6)	61(28.4)	0.66(0.37–1.15)	0.146
More than 7 times a week	172(37.8)	135(78.4)	37(21.6)	0.45(0.24–0.83)	0.011*
**Stay with animals**					
Yes	16 (3.3)	14(87.5)	2(12.5)	1.00	
No	474 (96.7)	346(73)	128(27)	2.59(0.60–11.55)	0.212
**Drainage**					
Well drained	423(86)	313(74)	110(26)	1.00	
Poorly drained	69(14)	49(71)	20(29)	1.16(0.66–2.04)	0.603

*level of significance at p<0.05,

**level of significance at p<0.001, p<0.05

### Predictors of diarrhea disease among children under five years in Lira City Northern Uganda at Multivariate logistic regression analysis

Table 6 shows that factors associated with diarrhea disease in the multivariate logistic regression analysis were children between 49–60 months old (AOR = 0.11, 95% *CI*: 0.03–0.39, P = 0.001), cleaning the latrine more than 7 times in a week (AOR = 0.42, 95% *CI*: 0.22–0.81, P = 0.010) and those who did not treat their drinking water (AOR = 1.84, 95% *CI*: 1.11–3.06, P = 0.018. Children between 49 to 60 months were 89% less likely to experience episodes of diarrhea compared to those who were 1–12 months. Children whose households cleaned their latrines more than 7 times in a week were 58% less likely to experience diarrhea episodes compared to those whose households cleaned their latrines less than 7 times a week. Lastly, children in households that did not treat their drinking water were 1.84 times more likely to experience diarrhea episodes compared to those in households that treated their drinking water.

**Table 6:** Associated factors of diarrheal disease among children under five years in Lira City Northern Uganda.

Variable	COR (*95%CI*)	P value	AOR (*95%CI*)	P value
**Age of child**				
1–12 months	1.00		1.00	
13–24 months	0.65(0.37–1.15)	0.163	0.65(0.36–1.18)	0.163
24–36 months	0.63(0.36–1.07)	0.026	0.52(0.29–0.92)	0.026*
37–48 months	0.51(0.27–0.95)	0.023	0.46(0.24–0.90)	0.023*
49–60 months	0.12(0.04–0.42)	0.001**	0.11(0.03–0.39)	0.001**
**Family size**				
1–5	1.00		1.00	
6–10	1.32(0.86–2.03)	0.199	1.50(0.94–2.39)	0.089
>11	4.8(1.31–17.42)	0.17	2.98(0.72–12.24)	0.130
**Employment status**				
Employed	1.00		1.00	
Self employed	0.52(0.25–1.09)	0.086	0.52(0.23–1.21)	0.130
Unemployed	0.63(0.31–1.25)	0.188	0.54(0.24–1.19)	0.129
**Number of times latrine is cleaned**				
Less than 7 times a week	1.00		1.00	
7 times a week (every day)	0.655(0.37–1.15)	0.146	0.63(0.34–1.16)	0.141
More than 7 times a week	0.45(0.24–0.83)	0.011**	0.42(0.22–0.81)	0.010**
**Water treatment**				
Boiling	1.00		1.00	
Filtration	1.04(0.36–3.00)	0.937	0.88(0.29–2.60)	0.826
Chemical treatment	1.22(0.58–5.37)	0.316	1.59(0.49–5.12)	0.432
No treatment	1.19(01.29–3.41)	0.005**	1.84(1.11–3.06)	0.018**

*Level of significance at p<0.05,

**level of significance at p<0.001, p<0.05

## Discussion

The present study aimed at determining the prevalence and assess the water, sanitation and hygiene risk factors associated with diarrheal diseases among children under five years in Lira City. Our results showed that 26.4% of the children under the age of five years had experienced episodes of diarrheal with in the previous two weeks. Factors associated with diarrheal disease were age of the child (AOR = 0.11, 95% *CI*: 0.03–0.39, P = 0.001), cleaning the latrine (AOR = 0.42, 95% *CI*: 0.22–0.81, P = 0.010) and use of untreated drinking water (AOR = 1.84, 95% *CI*: 1.11–3.06, P = 0.018).

This study reveals that the prevalence of diarrhea disease among children under five years in Lira City was 26.4%. This could be attributable to the lack of basic amenities for proper health and considerable independence where children play unsupervised within the community environment which is susceptible to contamination [17]. The 26.4% observed in this study is higher than the 20% and 20.5% national prevalence and Lango sub region prevalence respectively [15]. However, our result is lower than 29.1% reported in Pader and 62.4% in Wakiso districts [17, 18]. The variations observed among the studies may be attributed to differences in the settings where they were conducted. Lira City, situated in Northern Uganda, is characterized by limited access to Water, Sanitation, and Hygiene (WASH) facilities, particularly in urban centers. In contrast, areas like Pader district are positioned in resource-constrained regions marked by exceptionally high poverty levels. Additionally, Wakiso district, characterized by overcrowding and a substantial number of slums, presents elevated risk factors for diarrheal diseases.

Our finding in this study show that, older children aged 49–60 months were 89% less likely to suffer diarrhea than their younger peers. This may not be a surprising result because children under the age of 11 months have a comparatively weaker immune system compared to their older counterparts [28]. Moreover, younger children who are learning to crawl and walk are exposed to a variety of diarrhea risk factors [29]. Our results are consistent with other previous studies conducted in Africa [29, 30].

Our results indicate that children whose caregivers cleaned their latrines more than seven times per week were 58% less likely to contract diarrhea. This is because disposing of human excreta is critical for interrupting the cycle of diarrheal illness transmission [31]. Furthermore, maintaining the places where excreta is disposed of clean guarantees that no diarrhea-causing substances are transferred back to the children, resulting in diarrheal illness [32]. This finding is similar to previous studies that emphasizes good sanitation in WASH facilities to prevent diarrheal disease in children under the age of 5 years [33]. This means that, while correct disposal of human excreta is critical for preventing diarrheal disease, the sites where the excreta is disposed should be kept clean to avoid the potential of transmission.

Our study also revealed that children who lived in households that did not treat their drinking water were 1.84 times more likely to have diarrhea episodes compared to children whose families treated the water. This is due to the fact that ingesting microorganisms that could cause diarrhea are considerably reduced following water treatment. This view is supported by Soboksa and colleagues, who argue that collecting water from improved water sources does not necessarily lessen the incidence of diarrhea. Additionally, drinking water must be treated [34]. This result is consistent with other studies indicating that water treatment is important in avoiding diarrheal disease in children [25, 35, 36].

### Study limitations

The prevalence of diarrhea and WASH-associated factors relies heavily on self-reporting by parents or caregivers, which could be susceptible to recall bias or social desirability bias, leading to inaccuracies in the data. Furthermore, the study might not account for the complete health history of children, including previous illnesses or treatments, which could impact the prevalence of diarrhea. Also the study’s cross-sectional design may only provide a snapshot of the prevalence and associated factors, making it challenging to establish causation or capture changes over time.

## Conclusion

The study reveals that one in four children under five experienced diarrheas in the past two weeks, under scoring trends observed globally and locally. Factors such as the child’s age, latrine cleanliness, and water treatment emerged as crucial contributors to this prevalence. These findings also underscore the need for urgent and targeted interventions to reduce diarrhea among young children not only in Uganda’s emerging urban regions but also in comparable global contexts. The call for heightened sensitization efforts on latrine cleanliness and household water treatment aligns with successful strategies employed elsewhere, emphasizing the universal relevance of addressing these challenges in urban settings worldwide.

## Supporting information

S1 Data (DTA)

## Abbreviations

LCC: Lira City Council
MOH: Ministry of Health
UBOS: Uganda Bureau of Statistics
UNICEF: United Nations International Fund
WASH: Water Sanitation and Hygiene
WHO: World Health Organization

## References

[1] UNICEF. Water, Sanitation and Hygiene (WASH). 2022 [cited 8 Dec 2022]. https://www.unicef.org/wash

[2] Vos T, Barber RM, Bell B, Bertozzi-Villa A, Biryukov S, Bolliger I, et al. Global, regional, and national incidence, prevalence, and years lived with disability for 301 acute and chronic diseases and injuries in 188 countries, 1990–2013: a systematic analysis for the Global Burden of Disease Study 2013. The Lancet. 2015;386: 743–800. doi: 10.1016/S0140-6736(15)60692-4 26063472 PMC4561509

[3] Kotloff KL, Platts-Mills JA, Nasrin D, Roose A, Blackwelder WC, Levine MM. Global burden of diarrheal diseases among children in developing countries: Incidence, etiology, and insights from new molecular diagnostic techniques. Vaccine. 2017;35: 6783–6789. doi: 10.1016/j.vaccine.2017.07.036 28765005

[4] WHO. Diarrhoea. 2022 [cited 7 Dec 2022]. https://www.who.int/health-topics/diarrhoea

[5] Mokomane M, Kasvosve I, de Melo E, Pernica JM, Goldfarb DM. The global problem of childhood diarrhoeal diseases: emerging strategies in prevention and management. Ther Adv Infect Dis. 2018;5: 29–43. doi: 10.1177/2049936117744429 29344358 PMC5761924

[6] Troeger C, Blacker BF, Khalil IA, Rao PC, Cao S, Zimsen SR, et al. Estimates of the global, regional, and national morbidity, mortality, and aetiologies of diarrhoea in 195 countries: a systematic analysis for the Global Burden of Disease Study 2016. Lancet Infect Dis. 2018;18: 1211–1228. doi: 10.1016/S1473-3099(18)30362-1 30243583 PMC6202444

[7] Agaro A, Hareru HE, Muche T, Sisay W/tsadik D, Ashuro Z, Negassa B, et al. Predictors of Hand-Washing Practices at Critical Times Among Mothers of Under-5 Years Old Children in Rural Setting of Gedeo Zone, Southern Ethiopia. Environ Health Insights. 2022;16: 11786302221120784. doi: 10.1177/11786302221120784 36051946 PMC9425877

[8] Solomon ET, Gari SR, Kloos H, Alemu BM. Handwashing effect on diarrheal incidence in children under 5 years old in rural eastern Ethiopia: a cluster randomized controlled trial. Trop Med Health. 2021;49: 26. doi: 10.1186/s41182-021-00315-1 33757600 PMC7989202

[9] Hailu B, Ji-Guo W, Hailu T. Water, Sanitation, and Hygiene Risk Factors on the Prevalence of Diarrhea among Under-Five Children in the Rural Community of Dangila District, Northwest Ethiopia. J Trop Med. 2021;2021. doi: 10.1155/2021/2688500 34745270 PMC8564202

[10] Jeyakumar A, Godbharle SR, Giri BR. Water, sanitation and hygiene (WaSH) practices and diarrhoea prevalence among children under five years in a tribal setting in Palghar, Maharashtra, India. J Child Health Care Prof Work Child Hosp Community. 2021;25: 182–193. doi: 10.1177/1367493520916028 32249584

[11] Zerbo A, Castro Delgado R, Arcos González P. Water sanitation and hygiene in Sub-Saharan Africa: Coverage, risks of diarrheal diseases, and urbanization. J Biosaf Biosecurity. 2021;3: 41–45. doi: 10.1016/j.jobb.2021.03.004

[12] Pirozzi G. Progress on household drinking water, sanitation and hygiene I 2000–2017 Progress on household drinking water, sanitation and hygiene I 2000–2017 Progress on household drinking water, sanitation and hygiene 2000–2017: Special focus on inequalities. 2016.

[13] MOH. Uganda Sanitation Fund Programme Annual Report for Financial Year 2013 / 14. 2014.

[14] Ssemugabo C, Wafula ST, Ndejjo R, Osuret J, Musoke D, Halage AA. Characteristics of sanitation and hygiene facilities in a slum community in Kampala, Uganda. Int Health. 2021;13: 13–21. doi: 10.1093/inthealth/ihaa011 32236413 PMC7807239

[15] UBOS. Uganda Demographic and Health Survey 2016. Udhs 2016. 2016; 625–625. www.DHSprogram.com

[16] Nambuusi BB, Ssempiira J, Makumbi FE, Kasasa S, Vounatsou P. The effects and contribution of childhood diseases on the geographical distribution of all-cause under-five mortality in Uganda. Parasite Epidemiol Control. 2019;5. doi: 10.1016/j.parepi.2019.e00089 30923753 PMC6424012

[17] Nantege R, Kajoba D, Ddamulira C, Ndoboli F, Ndungutse D. Prevalence and factors associated with diarrheal diseases among children below five years in selected slum settlements in Entebbe municipality, Wakiso district, Uganda. BMC Pediatr. 2022;22: 394. doi: 10.1186/s12887-022-03448-2 35799163 PMC9264597

[18] Omona S, Malinga GM, Opoke R, Openy G, Opiro R. Prevalence of diarrhoea and associated risk factors among children under five years old in Pader District, northern Uganda. BMC Infect Dis. 2020;20: 37. doi: 10.1186/s12879-020-4770-0 31931735 PMC6958783

[19] UNICEF. Government launches new rotavirus vaccine to protect children from diarrhea. 2018 [cited 19 Nov 2023]. https://www.unicef.org/uganda/press-releases/government-launches-new-rotavirus-vaccine-protect-children-diarrhea

[20] Finnström S. War Stories and Troubled Peace. Curr Anthropol. 2015;56: S222–S230. doi: 10.1086/683270

[21] LCC. LIRA CITY COUNCIL–Lira City Council. 2021 [cited 8 Dec 2022]. https://liracitycouncil.go.ug/

[22] UBOS. Nation population and Housing census area profiles 2014. Lira District; 2017.

[23] Kish L. Sampling organizations and groups of unequal sizes. Am Sociol Rev. 1965; 564–572. 14325826

[24] Ahmad R, Farooq U, Ali D, Shah AQ. Alternative care takers for children of health care workers during COVID-19 pandemic in Kashmir valley: a cross sectional study. Int J Community Med Public Health. 2023;10: 635. https://www.researchgate.net/profile/Ab-Qayoom/publication/368389058_Alternative_care_takers_for_children_of_health_care_workers_during_COVID-19_pandemic_in_Kashmir_valley_a_cross_sectional_study/links/63e5c6de64252375639f8aba/Alternative-care-takers-for-children-of-health-care-workers-during-COVID-19-pandemic-in-Kashmir-valley-a-cross-sectional-study.pdf

[25] Solomon ET, Robele S, Kloos H, Mengistie B. Effect of household water treatment with chlorine on diarrhea among children under the age of five years in rural areas of Dire Dawa, eastern Ethiopia: a cluster randomized controlled trial. Infect Dis Poverty. 2020;9: 64. doi: 10.1186/s40249-020-00680-9 32513277 PMC7278122

[26] UNHCR. WASH needs assessment—UNHCR|Emergency Handbook. 2018 [cited 19 Dec 2022]. https://emergency.unhcr.org/entry/38439/wash-needs-assessment

[27] UNICEF. UNICEF, WHO Issue Survey Questions for Monitoring WASH | News | SDG Knowledge Hub | IISD. 2018. https://sdg.iisd.org:443/news/unicef-who-issue-survey-questions-for-monitoring-wash/

[28] Simon AK, Hollander GA, McMichael A. Evolution of the immune system in humans from infancy to old age. Proc R Soc B Biol Sci. 2015;282: 20143085. doi: 10.1098/rspb.2014.3085 26702035 PMC4707740

[29] Mosisa D, Aboma M, Girma T, Shibru A. Determinants of diarrheal diseases among under five children in Jimma Geneti District, Oromia region, Ethiopia, 2020: a case-control study. BMC Pediatr. 2021;21: 532. doi: 10.1186/s12887-021-03022-2 34847912 PMC8630872

[30] Tareke AA, Enyew EB, Takele BA. Pooled prevalence and associated factors of diarrhea among under-five years children in East Africa: A multilevel logistic regression analysis. PLOS ONE. 2022;17: e0264559. doi: 10.1371/journal.pone.0264559 35421129 PMC9009646

[31] WHO. Diarrhoeal disease. 2017 [cited 19 May 2023]. https://www.who.int/news-room/fact-sheets/detail/diarrhoeal-disease

[32] Majorin F, Torondel B, Ka Seen Chan G, Clasen T. Interventions to improve disposal of child faeces for preventing diarrhoea and soil-transmitted helminth infection. Cochrane Database Syst Rev. 2019;2019: CD011055. doi: 10.1002/14651858.CD011055.pub2 31549742 PMC6757260

[33] Agustina, Dukabain OM, Singga S, Wanti W, Suluh DG, Mado FG. Home sanitation facilities and prevalence of diarrhea for children in Oelnasi Village, Kupang Tengah Sub-district. Gac Sanit. 2021;35: S393–S395. doi: 10.1016/j.gaceta.2021.10.059 34929859

[34] Soboksa NE. Associations Between Improved Water Supply and Sanitation Usage and Childhood Diarrhea in Ethiopia: An Analysis of the 2016 Demographic and Health Survey. Environ Health Insights. 2021;15. doi: 10.1177/11786302211002552 33795933 PMC7975481

[35] Wolf J, Hubbard S, Brauer M, Ambelu A, Arnold BF, Bain R, et al. Effectiveness of interventions to improve drinking water, sanitation, and handwashing with soap on risk of diarrhoeal disease in children in low-income and middle-income settings: a systematic review and meta-analysis. The Lancet. 2022;400: 48–59. doi: 10.1016/S0140-6736(22)00937-0 35780792 PMC9251635

[36] Komarulzaman A, Smits J, de Jong E. Clean water, sanitation and diarrhoea in Indonesia: Effects of household and community factors. Glob Public Health. 2017;12: 1141–1155. doi: 10.1080/17441692.2015.1127985 26758565

